# Bioequivalence of budesonide inhalation suspension with and without activated charcoal in healthy Chinese subjects: two randomized four-period crossover trials

**DOI:** 10.3389/jpps.2026.17048

**Published:** 2026-10-20

**Authors:** Yinjuan Li, Lu Qi, Rongxia Fan, Qian Zhang, Ju Liu, Xiaoqiang Cheng, Xinghe Wang

**Affiliations:** 1 Department of Phase I Clinical Trial Center, Beijing Shijitan Hospital, Capital Medical University, Beijing, China; 2 Department of Allergy, Beijing Shijitan Hospital, Capital Medical University, Beijing, China; 3 Special Medical Services Department, Beijing Shijitan Hospital, Capital Medical University, Beijing, China

**Keywords:** activated charcoal, bioequivalence, budesonide inhalation suspension, pharmacokinetics, pulmonary absorption

## Abstract

**Background:**

Demonstrating bioequivalence for locally acting inhaled products is challenging because systemic exposure may reflect both pulmonary absorption and gastrointestinal absorption of the swallowed fraction. Activated charcoal can suppress gastrointestinal uptake and may help interpret lung-derived exposure more directly.

**Methods:**

Two open-label, randomized, single-dose, four-period crossover pharmacokinetic bioequivalence trials were conducted in healthy Chinese adults, one under non-block conditions and one under charcoal-block conditions. Thirty-six subjects were enrolled in each trial. In each period, subjects inhaled a single 1 mg dose of the test or reference budesonide inhalation suspension via a standardized jet nebulizer procedure, with a 5-day washout between periods. Plasma budesonide concentrations were measured for 15 h. The primary pharmacokinetic endpoints were C_max_, AUC_0-t_, and AUC_0-inf_, and bioequivalence was concluded if the 90% confidence intervals for the test/reference geometric mean ratios were within 80.00%–125.00%.

**Results:**

Under both non-block and charcoal-block conditions, the 90% confidence intervals for C_max_, AUC_0-t_, and AUC_0-inf_ met the predefined bioequivalence criteria. In exploratory between-cohort comparisons based on subject-level arithmetic summaries, activated-charcoal blockade was associated with numerically lower AUC values, whereas C_max_ remained similar; however, nominal exploratory P values for C_max_, AUC_0-t_, and AUC_0-inf_ were all >0.05 and were not used for confirmatory inference. These findings are consistent with, but do not establish, a predominant pulmonary contribution to systemic exposure after nebulization. Both formulations were well tolerated, and no serious adverse events were reported.

**Conclusion:**

The test and reference budesonide inhalation suspension formulations were bioequivalent under both non-block and charcoal-block conditions. Exploratory between-cohort comparisons showed no statistically significant differences in C_max_, AUC_0-t_, or AUC_0-inf_ based on nominal P values. The charcoal-related findings are consistent with, but do not establish, a predominant pulmonary contribution to systemic exposure after nebulization and should be interpreted cautiously because the conditions were evaluated in separate cohorts. Overall, the two studies provide supportive pharmacokinetic evidence for the evaluation of generic nebulized budesonide products.

**Trial Registration:**

Clinical Trial Registration: Drug Clinical Trial Registration and Information Publicity Platform of the National Medical Products Administration of China, CTR20200344 and CTR20201473 (http://www.chinadrugtrials.org.cn/); Chinese Clinical Trial Registry, ChiCTR2000035173 (https://www.chictr.org.cn/).

## Introduction

Asthma and chronic obstructive pulmonary disease (COPD) are major chronic inflammatory airway diseases that continue to impose substantial burdens on public health, healthcare utilization, and quality of life worldwide [1, 2]. This burden is also considerable in China, where both diseases remain common and underrecognized in parts of the population [3, 4].

Inhaled corticosteroids (ICSs) are central to the long-term management of these conditions because they deliver anti-inflammatory therapy directly to the airways while limiting systemic exposure relative to oral corticosteroids [1, 2, 5]. Budesonide inhalation suspension (BIS), marketed as Pulmicort Respules, is a widely used nebulized ICS with established efficacy and safety. It is particularly useful for patients who cannot reliably use handheld inhalers, such as young children, older adults, and patients with poor inspiratory coordination or severe airflow limitation [6, 7].

Demonstrating bioequivalence (BE) for locally acting orally inhaled drug products is challenging because drug concentrations at the pulmonary site of action cannot be measured directly in routine clinical studies. Pharmacokinetic (PK) studies are therefore commonly used as surrogate evidence, but regulatory strategies differ across jurisdictions. Standard PK studies quantify total systemic exposure, which reflects both pulmonary absorption and gastrointestinal absorption after swallowing. For products such as BIS, activated charcoal blockade is a useful additional tool because it minimizes gastrointestinal absorption and enables a more direct assessment of pulmonary delivery [5, 8–11]. An alternative, still investigational approach has been proposed for orally inhaled products: the evaluation of post-inhalation exhaled breath condensate (EBC) concentration-time profiles using conventional BE metrics. In a pilot salbutamol study, EBC-derived C_max_ and AUC differentiated two inhaled formulations, suggesting that EBC may provide complementary information on airway exposure; however, larger confirmatory studies are required before this method can be considered established for regulatory BE assessment [9].

Despite this regulatory and methodological framework, published BIS studies that combine charcoal-block and non-block PK evaluations in Asian populations remain limited. Accordingly, we conducted two complementary PK-BE trials comparing a test BIS formulation manufactured by Sichuan Purity Pharmaceutical Co., Ltd. with the reference product Pulmicort Respules in healthy Chinese subjects. The non-block trial was designed to evaluate total systemic exposure, whereas the charcoal-block trial was designed to provide a lung-focused assessment by reducing gastrointestinal absorption. Together, the two trials were intended to support an integrated pharmacological and regulatory interpretation of BE for a generic nebulized corticosteroid product.

## Materials and methods

### Ethics approval, trial registration, and reporting standards

Both studies were conducted in accordance with Good Clinical Practice, the Declaration of Helsinki, and applicable national regulations. The protocols were reviewed and approved by the independent ethics committee of Beijing Shijitan Hospital, Capital Medical University. The non-block trial received ethical approval on November 13, 2019 (Approval No.: 2019 Lun Shen No. 116); the charcoal-block trial obtained ethical approval on April 29, 2020 (Approval No.: 2020 Lun Shen No. 13).

All participants provided written informed consent before any study-specific procedure and could withdraw at any time without prejudice.

The two trials were registered at the Drug Clinical Trial Registration and Information Publicity Platform of the National Medical Products Administration of China under identifiers CTR20200344 (first participant enrolled on December 23, 2019) and CTR20201473 (first participant enrolled on July 20, 2020). The charcoal-block trial was additionally entered in the Chinese Clinical Trial Registry under identifier ChiCTR2000035173 on August 2, 2020, as a supplementary public registration record. Accordingly, although the supplementary ChiCTR entry postdated first participant enrollment in the charcoal-block study, the study had already been registered in the mandatory NMPA drug-trial registry before enrollment. The clinical trial registration identifiers and public registry URLs are reported in the Abstract in accordance with journal requirements. Trial reporting was aligned with the Consolidated Standards of Reporting Trials (CONSORT) principles for randomized clinical trials, and participant disposition is summarized in Figure 1.

**FIGURE 1:**
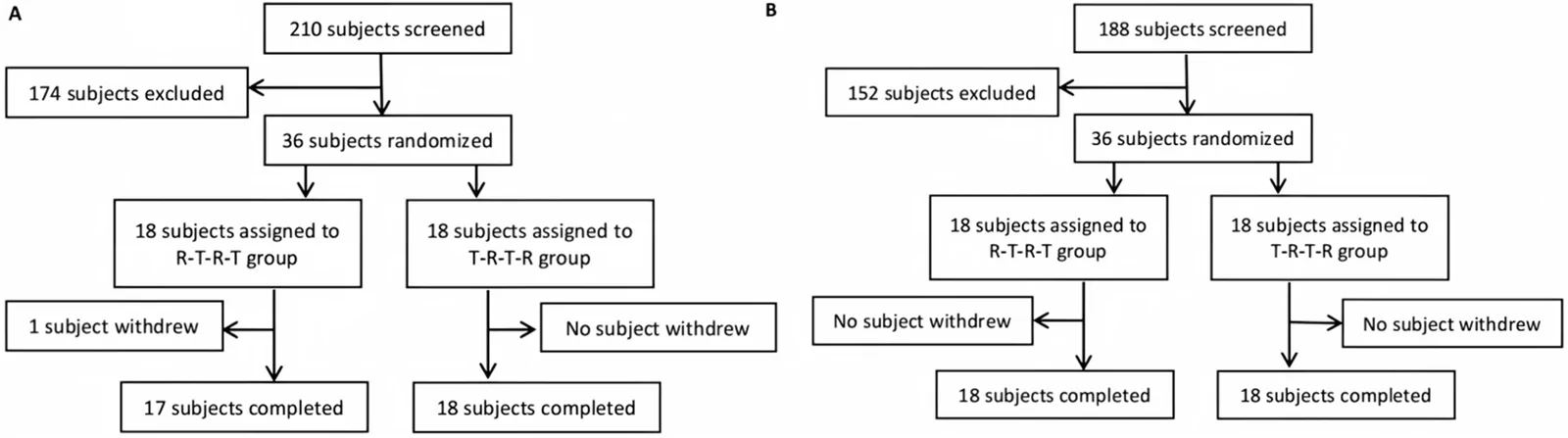
Participant disposition in the non-block and charcoal-block bioequivalence studies. **(A)** Non-block study. **(B)** Charcoal-block study.

### Study design

Two open-label, randomized, single-dose, four-period crossover PK-BE studies were performed under fasting conditions, one without activated charcoal and one with activated charcoal blockade. A 5-day washout interval separated consecutive study periods.

Sample size was estimated on the basis of pilot data and published variability estimates for inhaled budesonide [12]. In our pilot work, the intra-individual variation (IIV) for Cmax and AUC was approximately 25.0% and 19.0%, respectively. Sample size was determined based on the primary PK endpoints C_max_ and AUC, assuming α = 0.05, β = 0.20, IIV = 25%, assumed T/R mean ratios of 90%–111%, and BE limits of 80.00%–125.00%. Under these assumptions, 28 evaluable subjects were required; to allow for potential dropout, 36 subjects were enrolled in each study.

### Study drugs and nebulization equipment

The investigational products were two formulations of BIS. The test product was manufactured by Sichuan Purity Pharmaceutical Co., Ltd. (2 mL:1 mg per ampoule; batch No. CP024-201902; manufacture date May 19, 2019; expiry date May 18, 2021).

The reference product was Pulmicort Respules (AstraZeneca Pty Ltd; 2 mL:1 mg per ampoule). Batch No. 325707 was used in the charcoal-block study and batch No. 324560 in the non-block study, with expiry dates in September 2021 and February 2021, respectively.

Drug administration was performed with a PARI LC Jet Plus nebulizer connected to a PARI Master compressor. Before each period, all subjects received standardized nebulization training. During dosing, subjects sat upright, wore nasal clips to prevent nasal breathing, and inhaled slowly and steadily through a mouthpiece while following a standardized audio guide lasting approximately 6 min.

### Study population and drug administration

Eligible participants were healthy Chinese adults aged 18–65 years with a body mass index of 19.0–26.0 kg/m^2^. Eligibility was determined by medical history, physical examination, vital signs, lung function testing, 12-lead electrocardiography, and clinical laboratory evaluations. Women of childbearing potential were required to have a negative pregnancy test and to use reliable contraception; male participants were also required to use contraception during the study and for 3 months thereafter. Subjects with asthma, airway hyperresponsiveness, or oral conditions such as ulcers were excluded.

A total of 72 subjects were enrolled, with 36 subjects assigned to each study. Within each study, subjects were randomized in a 1:1 ratio to one of two treatment sequences (T-R-T-R or R-T-R-T), where T denotes the test formulation and R denotes the reference formulation. Randomization was generated using SAS version 9.4.

Subjects were admitted to the study center 2 days before dosing for nebulization training. Only those who completed training successfully were dosed. After an overnight fast of at least 10 h, each subject inhaled a single 1 mg dose of either T or R BIS via the study nebulizer. Nebulization continued until 6 min after sputtering was first heard.

In the charcoal-block study, activated charcoal (3 g suspended in 30 mL water) was administered 2 min before nebulization, immediately after nebulization, and 1 h after nebulization. Participants were instructed to gargle the suspension before swallowing to maximize adsorption of drug remaining in the oral cavity. All dosing procedures were conducted under the supervision of trained study personnel, with protocol-specific instruction before each dosing period. This procedure was designed to minimize gastrointestinal absorption and provide a lung-focused assessment of systemic exposure.

### Safety monitoring

Safety monitoring after each dose included physical examination, electrocardiography, vital signs, clinical laboratory testing, adverse event recording, and documentation of concomitant medication. The washout interval between periods was 5 days. Adverse event severity was graded according to Common Terminology Criteria for Adverse Events, version 5.0.

### Blood sampling and bioanalytical assay

Blood samples for PK analysis were collected at 1.5 h before administration and at 3, 5, 8, 10, 12, 16, 20, 25, 30, and 45 min and 1.0, 1.5, 2.0, 3.0, 6.0, 10.0, and 15.0 h after administration. The nominal sample volume at each time point was 4 mL.

Plasma samples were separated on a Waters Acquity UPLC BEH C_18_ column (2.1 mm × 50 mm, 1.7 μm) maintained at 40 °C, with a Phenomenex UHPLC C_18_ precolumn (2.1 mm internal diameter). The mobile phases were an acetic acid LC-MS solution in ultrapure water and methanol LC-MS grade solvent. The injection volume was 20 μL and the flow rate was 0.4 mL/min.

Quantification of budesonide and budesonide-d_8_ was performed by multiple reaction monitoring with electrospray ionization in negative mode. Optimized source parameters were as follows: GAS1 40 psi, GAS2 50 psi, curtain gas 30 psi, collision gas 8 psi, ion spray voltage −4,500 V, and source temperature 450 °C. The monitored ion transitions were m/z 489.3→357.1 for budesonide and m/z 497.3→357.0 for budesonide-d_8_. Declustering potential, collision energy, entrance potential, and collision cell exit potential were set according to validated analytical conditions.

Blood samples were centrifuged within 1 h of collection at 1,700 × g and 4 °C for 10 min to obtain plasma. The separated plasma was stored below −60 °C until analysis, within the stability window established during bioanalytical method validation.

Plasma budesonide concentrations were quantified using a validated LC-MS/MS assay in human K2-EDTA plasma. The calibration curve was linear over 10.00–2000 pg/mL, and the lower limit of quantification (LLOQ) was 10.00 pg/mL. During study-sample analysis, quality control (QC) concentrations were 30.00, 250.0, 800.0, and 1500 pg/mL, with a 6000 pg/mL dilution QC, and QC samples were distributed throughout each analytical run. QC precision ranged from 2.72% to 6.03% in the non-block study and from 1.99% to 3.98% in the charcoal-block study; corresponding QC bias ranged from −1.35% to 1.70% and from −1.07% to 7.25%, respectively. Plasma stability met acceptance criteria after four freeze-thaw cycles, 8 h on ice before processing, 72 h in the autosampler after processing, and 78 h of reinjection storage. Long-term plasma stability was demonstrated for 23 days at −30 °C to −10 °C and for 74 days at −50 °C to −30 °C and −80 °C to −60 °C. In the charcoal-block study, whole blood was additionally shown to be stable on ice for 2 h before plasma separation. The storage duration of clinical samples remained within these validated stability windows. Full validation data are provided in Supplementary Material S1.

### PK and BE analysis

PK parameters, including C_max_, AUC_0-t_, AUC_0-inf_, terminal elimination half-life, and T_max_, were calculated by noncompartmental analysis using Phoenix WinNonlin version 8.2. Actual blood sampling times were used for PK calculations. BE was evaluated in the bioequivalence set (BES). C_max_, AUC_0-t_, and AUC_0-inf_ were natural log-transformed and analyzed in SAS 9.4 using a mixed-effects model for the four-period fully replicated crossover design. Sequence, period, and treatment/formulation were included as fixed effects, and subjects were handled as the subject term nested within sequence in the replicated crossover framework. Geometric mean ratios and their 90% CIs were derived under the two one-sided testing approach; BE was concluded when the 90% confidence intervals (CIs) for C_max_, AUC_0-t_, and AUC_0-inf_ were entirely within 80.00%–125.00%. In the non-block study, one subject withdrew after completing periods 1 and 2; the evaluable data from these periods were retained in pharmacokinetic parameter set (PKPS)/BES without imputation.

For the exploratory between-cohort comparison, the two replicate administrations of each formulation were averaged within each participant. The resulting subject-level summaries were compared between the non-block and charcoal-block cohorts separately for the test and reference formulations using two-sided Welch unequal-variance tests on the arithmetic scale. T_max_ was summarized descriptively. No adjustment for multiplicity was performed; therefore, all P values from these comparisons are reported as nominal exploratory P values and were not used for confirmatory inference.

## Results

### Subject disposition and baseline characteristics

Overall, 210 subjects were screened for the non-block study and 188 for the charcoal-block study (Figure 1). A total of 72 subjects were enrolled across the two studies. In the non-block study, 36 subjects were enrolled and 35 completed all four periods. One subject (K033) withdrew after period 2 because of an adverse event unrelated to study treatment; because dosing and PK sampling for periods 1 and 2 had been completed, this subject contributed evaluable period-1 and period-2 data to pharmacokinetic concentration set (PKCS), PKPS, and BES. In the charcoal-block study, all 36 enrolled subjects completed all four periods.

Demographic characteristics are summarized in Table 1. In the charcoal-block study, the mean age was 33.36 ± 6.89 years, the mean body weight was 61.45 ± 8.16 kg, and the mean body mass index was 22.81 ± 1.78 kg/m^2^. In the non-block study, the corresponding values were 37.17 ± 6.36 years, 63.79 ± 7.43 kg, and 23.71 ± 1.60 kg/m^2^. Male subjects accounted for 44.4% of the charcoal-block cohort and 61.1% of the non-block cohort.

**TABLE 1:** Demographic and baseline characteristics of enrolled subjects in the non-block and charcoal-block studies.

Parameters	Non-block	Charcoal-block
N	36	36
Gender n (%) Male Female	22 (61.1%) 14 (38.9%)	16 (44.4%) 20 (55.6%)
Age (y) Mean ± SD Min-max	37.17 ± 6.36 22–48	33.36 ± 6.89 21–45
Weight (kg) Mean ± SD Min-max	63.79 ± 7.43 50.9–78.4	61.45 ± 8.16 47.0–81.8
BMI (kg/m^2^) Mean ± SD Min-max	23.71 ± 1.60 20.0–25.9	22.81 ± 1.78 19.2–25.9

BMI indicates body mass index; Max, maximum; Min, minimum.

### PK profile

Detailed PK parameters are summarized in Table 2. Budesonide was absorbed rapidly under both study conditions, with a median T_max_ of approximately 0.20 h. Plasma concentrations then declined promptly, and the terminal half-life was approximately 3 h, consistent with rapid pulmonary absorption followed by systemic elimination (Figures 2, 3). In Figures 2B, 3B, the 0–3 h interval is presented on an expanded linear scale to facilitate visualization of the early absorption phase.

**TABLE 2:** Pharmacokinetic parameters of budesonide after administration of test and reference budesonide inhalation suspension formulations.

Parameters	Non-block		Charcoal-block	
	Test (n = 71)^a^	Reference (n = 71)^a^	Test (n = 72)	Reference (n = 72)
T_max_ (h)	0.20 (0.08,0.42) (40.49)	0.20 (0.05,0.50) (50.44)	0.20 (0.08,0.42) (40.35)	0.20 (0.08,0.50) (48.29)
C_max_ (pg/mL)	1147.51 ± 434.20 (37.84)	1189.55 ± 498.39 (41.90)	1126.53 ± 448.52 (39.81)	1228.52 ± 577.06 (46.97)
AUC_0-t_ (h·pg/mL)	2,147.81 ± 650.64 (30.29)	2,201.04 ± 752.32 (34.18)	1927.92 ± 612.83 (31.79)	2077.76 ± 588.90 (28.34)
AUC_0-inf_ (h·pg/mL)	2,236.36 ± 669.65 (29.94)	2,288.22 ± 780.85 (34.12)	2000.58 ± 621.20 (31.05)	2,155.25 ± 590.30 (27.39)
t_1/2_ (h)	3.19 ± 0.75 (23.34)	3.12 ± 0.75 (24.16)	2.85 ± 0.74 (26.09)	2.88 ± 0.64 (22.36)

T_max_, time to maximum concentration.

C_max_, maximum concentration.

AUC_0-t_ indicates area under the concentration-time curve from 0 to t.

AUC_0-inf_, area under the concentration-time curve from 0 to infinity.

t_1/2_, half-life of elimination.

CV, coefficient of variation.

All data are expressed as arithmetic mean ± standard deviation, except for T_max_, which was presented as median (minimum–maximum), followed by CV%.

^a^ One subject withdrew from the study after completing the second period.

n represents the number of evaluable treatment-period PK, profiles rather than the number of individual participants.

**FIGURE 2:**
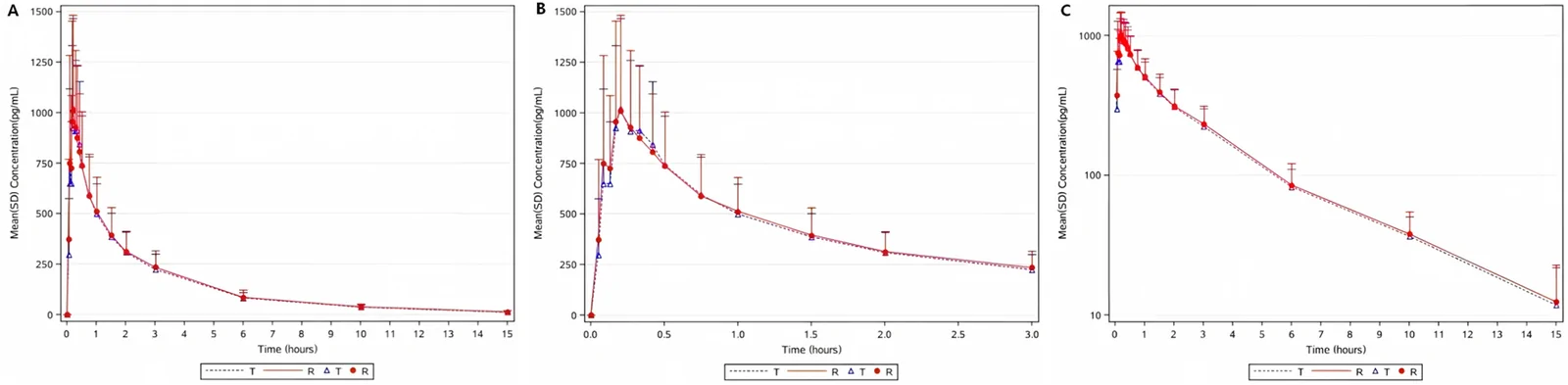
Mean plasma concentration-time profiles of budesonide after administration of the test and reference formulations under non-block conditions. **(A)** Linear scale (0–15 h). **(B)** Linear scale (0–3 h). **(C)** Semi-logarithmic scale (0–15 h).

**FIGURE 3:**
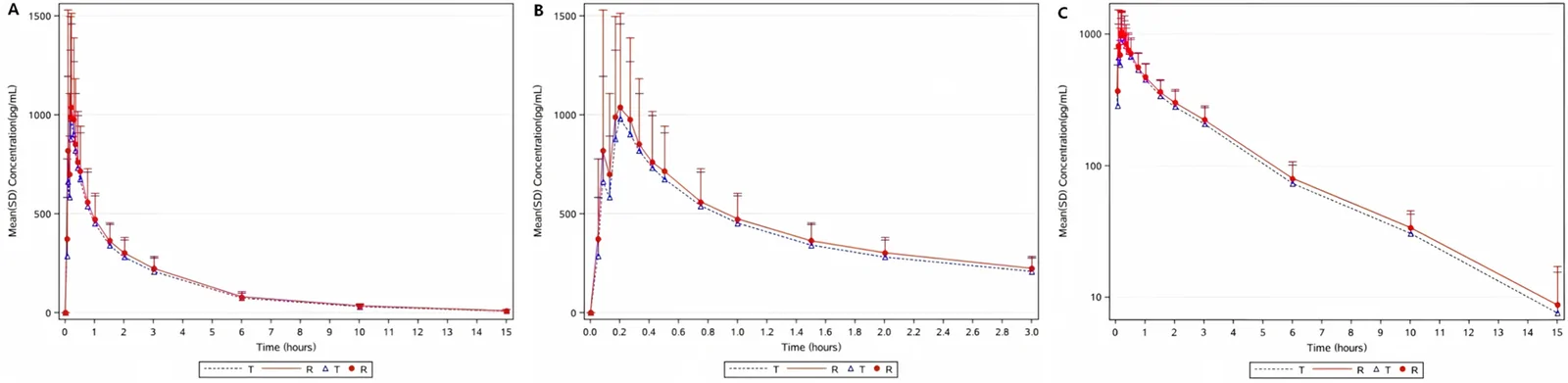
Mean plasma concentration-time profiles of budesonide after administration of the test and reference formulations under charcoal-block conditions. **(A)** Linear scale (0–15 h). **(B)** Linear scale (0–3 h). **(C)** Semi-logarithmic scale (0–15 h).

Table 3 presents an exploratory between-study comparison based on subject-level PK summaries derived from replicate administrations of the same formulation within each subject. Because the non-block and charcoal-block conditions were studied in separate cohorts, this analysis should be interpreted as exploratory only.

**TABLE 3:** Exploratory subject-level comparison of pharmacokinetic parameters under non-block and charcoal-block conditions.

Formulation	Parameter	Non-block N = 36	Charcoal-block N = 36	Mean difference (charcoal-block minus non-block)	Exploratory P value
Test	T_max_ (h)	0.200 (0.080, 0.375)	0.225 (0.083, 0.417)	—	Descriptive only
	C_max_ (pg/mL)	1142.76 ± 393.10	1126.53 ± 406.71	−16.23	0.864
	AUC_0-t_ (h·pg/mL)	2,135.20 ± 598.52	1927.92 ± 543.73	−207.28	0.129
	AUC_0-inf_ (h·pg/mL)	2,224.24 ± 613.06	2000.58 ± 550.29	−223.67	0.108
	t_1/2_ (h)	3.19 ± 0.61	2.85 ± 0.58	−0.35	0.016
Reference	T_max_ (h)	0.200 (0.065, 0.460)	0.200 (0.083, 0.500)	—	Descriptive only
	C_max_ (pg/mL)	1187.12 ± 454.98	1228.52 ± 518.03	41.41	0.720
	AUC_0-t_ (h·pg/mL)	2,190.83 ± 707.19	2077.76 ± 562.82	−113.08	0.456
	AUC_0-inf_ (h·pg/mL)	2,277.65 ± 736.99	2,155.25 ± 565.65	−122.40	0.432
	t_1/2_ (h)	3.12 ± 0.61	2.88 ± 0.52	−0.24	0.071

P values are nominal exploratory P values from two-sided Welch unequal-variance tests and were not adjusted for multiplicity. They were not used for confirmatory inference.

Values for C_max_, AU_C0-t_, AUC_0-inf_, and t_1/2_ are arithmetic mean ± SD, of subject-level summaries. T_max_ is reported as median (minimum, maximum). Each subject-level summary was calculated from the available replicate administrations of the same formulation within the same study. One non-block subject contributed 1 T period and 1 R period only after discontinuation following period 2.

Median T_max_ values were similar between conditions for both formulations. For the test formulation, mean C_max_ was 1142.76 ± 393.10 pg/mL under non-block conditions and 1126.53 ± 406.71 pg/mL under charcoal-block conditions, whereas mean AUC_0-t_ values were 2,135.20 ± 598.52 and 1927.92 ± 543.73 h·pg/mL and mean AUC_0-inf_ values were 2,224.24 ± 613.06 and 2000.58 ± 550.29 h·pg/mL, respectively. On the arithmetic scale, the nominal exploratory P values were 0.864 for C_max_, 0.129 for AUC_0-t_, and 0.108 for AUC_0-inf_, with mean differences (charcoal-block minus non-block) of −16.23, −207.28, and −223.67, respectively. For the reference formulation, the corresponding nominal exploratory P values were 0.720 for C_max_, 0.456 for AUC_0-t_, and 0.432 for AUC_0-inf_, with mean differences of 41.41, −113.08, and −122.40, respectively. Thus, activated charcoal was associated with numerically lower AUC values, whereas the between-condition differences in C_max_, AUC_0-t_, and AUC_0-inf_ were not statistically significant for either formulation. The shorter t_1/2_ observed for the test formulation under charcoal-block conditions (nominal P = 0.016) should be considered descriptive because no multiplicity adjustment was applied.

### BE evaluation

In the non-block study, all 36 randomized subjects were included in the BE set, although subject K033 contributed data only from the first two periods because of withdrawal after period 2. In the charcoal-block study, all 36 randomized subjects completed the BE evaluation.

As shown in Tables 4, 5, the 90% CIs for the geometric mean ratios of C_max_, AUC_0-t_, and AUC_0-inf_ all fell within the accepted BE range of 80.00%–125.00% under both non-block and charcoal-block conditions.

**TABLE 4:** Bioequivalence evaluation under non-block conditions.

PK parameters	GM and GMR			IIV%	90% CI	Power%
	T (n = 71)^a^	R (n = 71)^a^	GMR (T/R)%			
C_max_ (pg/mL)	1059.30	1080.00	98.08	26.17	91.41–105.24	99.91
AUC_0-t_ (h·pg/mL)	2038.50	2072.55	98.36	17.77	93.40–103.57	>99.99
AUC_0-inf_ (h·pg/mL)	2,129.17	2,157.13	98.70	17.18	93.89–103.77	>99.99

CI, confidence interval; GM, geometric mean; GMR, geometric mean ratio; IIV, intraindividual variation.

^a^ One subject withdrew after completing the second period.

**TABLE 5:** Bioequivalence evaluation under charcoal-block conditions.

PK parameters	GM and GMR			IIV%	90% CI	Power%
	T (n = 72)	R (n = 72)	GMR (T/R) %			
C_max_ (pg/mL)	1040.59	1111.59	93.61	24.83	86.73–101.04	96.12
AUC_0-t_ (h·pg/mL)	1821.71	1989.29	91.58	12.60	87.59–95.74	99.96
AUC_0-inf_ (h·pg/mL)	1896.66	2069.50	91.65	11.65	87.87–95.59	99.99

CI, confidence interval; GM, geometric mean; GMR, geometric mean ratio; IIV, intraindividual variation.

Analysis of log-transformed primary PK parameters (Table 6) showed no significant sequence effect in either study. In the non-block study, no significant formulation or period effects were identified. In the charcoal-block study, period effects were observed for C_max_, AUC_0-t_, and AUC_0-inf_, and formulation effects were observed for AUC_0-t_ and AUC_0-inf_. These analysis-of-variance findings did not alter the BE conclusion because the predefined decision criterion was based on the 90% CIs for the geometric mean ratios, all of which remained within the acceptance interval.

**TABLE 6:** Analysis of variance for log-transformed primary pharmacokinetic parameters.

Parameters	P (non-block)			P (charcoal-block)		
	LnC_max_	LnAUC_0-t_	LnAUC_0-inf_	LnC_max_	LnAUC_0-t_	LnAUC_0-inf_
Sequence	0.7005	0.8066	0.7938	0.8080	0.8665	0.8496
Period	0.2566	0.0840	0.0837	<0.0001	0.0010	0.0004
Formulation	0.6451	0.5914	0.6621	0.1531	0.0014	0.0009

ANOVA, analysis of variance. Bioequivalence was determined from the 90% confidence intervals for the geometric mean ratios, rather than from individual ANOVA P values.

Median T_max_ values were similar between the test and reference formulations in both studies. Signed-rank testing showed no statistically significant between-formulation difference in T_max_.

### Safety and tolerability

Safety analyses included all 72 subjects. Treatment-emergent adverse events are summarized in Tables 7, 8.

**TABLE 7:** Summary of treatment-emergent adverse events in the non-block study.

Parameters	Test (N = 36)		Reference (N = 36)		Total (N = 36)	
	n	N (%)	n	N (%)	n	N (%)
Total	7	6 (16.67%)	7	5 (13.89%)	14	10 (27.78%)
Upper respiratory tract infection	1	1 (2.78%)	0	0 (0%)	1	1 (2.78%)
Monocyte count increased	0	0 (0%)	1	1 (2.78%)	1	1 (2.78%)
Urine leukocyte esterase positive	1	1 (2.78%)	0	0 (0%)	1	1 (2.78%)
Elevated urinary bilirubin	0	0 (0%)	1	1 (2.78%)	1	1 (2.78%)
Urine ketone body positive	0	0 (0%)	1	1 (2.78%)	1	1 (2.78%)
Hematuria	1	1 (2.78%)	1	1 (2.78%)	2	2 (5.56%)
Decreased systolic blood pressure	1	1 (2.78%)	1	1 (2.78%)	2	1 (2.78%)
Elevated diastolic blood pressure	2	2 (5.56%)	0	0 (0%)	2	2 (5.56%)
Elevated blood bilirubin	1	1 (2.78%)	1	1 (2.78%)	2	2 (5.56%)
Elevated platelet count	0	0 (0%)	1	1 (2.78%)	1	1 (2.78%)

N (%), number (percentage) of subjects with adverse events; n, number of adverse events.

**TABLE 8:** Summary of treatment-emergent adverse events in the charcoal-block study.

Parameters	Test (N = 36)		Reference (N = 36)		Total (N = 36)	
	n	N (%)	n	N (%)	n	N (%)
Total	7	5 (13.89%)	4	4 (11.11%)	11	9 (25.00%)
Urine ketone body positive	1	1 (2.78%)	1	1 (2.78%)	2	2 (5.56%)
Blood unconjugated bilirubin increased	1	1 (2.78%)	0	0 (0%)	1	1 (2.78%)
Decreased systolic blood pressure	0	0 (0%)	1	1 (2.78%)	1	1 (2.78%)
Elevated alanine aminotransferase	0	0 (0%)	1	1 (2.78%)	1	1 (2.78%)
Upper respiratory tract infection	1	1 (2.78%)	0	0 (0%)	1	1 (2.78%)
Folliculitis	0	0 (0%)	1	1 (2.78%)	1	1 (2.78%)
Nausea	1	1 (2.78%)	0	0 (0%)	1	1 (2.78%)
Chest tightness	1	1 (2.78%)	0	0 (0%)	1	1 (2.78%)
Headache	1	1 (2.78%)	0	0 (0%)	1	1 (2.78%)
Hypokalemia	1	1 (2.78%)	0	0 (0%)	1	1 (2.78%)

N (%), number (percentage) of subjects with adverse events; n, number of adverse events.

In the non-block study, 12 subjects (33.33%) reported 20 adverse events, and 10 subjects (27.78%) experienced 14 treatment-emergent adverse events. One subject withdrew because of a grade 2 upper respiratory tract infection; all remaining adverse events were grade 1, and no serious adverse events occurred.

In the charcoal-block study, 11 subjects (30.56%) reported 13 adverse events, and 9 subjects (25.00%) experienced 11 treatment-emergent adverse events. One subject experienced grade 2 chest tightness approximately 2 h after the last dose; this symptom resolved quickly following administration of nasal cannula oxygen therapy; all other adverse events were grade 1. No serious adverse events or adverse-event-related withdrawals occurred, and all events had resolved by the final visit.

The incidence and pattern of adverse events were similar between the test and reference formulations, and the observed safety profile was consistent with the known tolerability of inhaled budesonide [7].

## Discussion

The present work shows that the test and reference BIS formulations were bioequivalent in healthy Chinese subjects under both non-block and charcoal-block conditions. Because the two trials were conducted with the same crossover framework, standardized nebulization procedure, and similar sampling schedule, the findings provide complementary evidence regarding total systemic exposure and lung-focused exposure for a generic nebulized corticosteroid product.

The overall PK profile observed here was consistent with previous reports for inhaled budesonide, characterized by rapid absorption, early peak concentrations, and an elimination half-life of approximately 3 h [13–17]. Although cross-study comparisons should be interpreted cautiously because inhalation device performance, inhalation technique, and analytical schedules differ across studies, the present data do not suggest any unexpected disposition pattern in this Chinese population.

The charcoal-block trial adds an informative mechanistic dimension to the BE assessment. In the exploratory between-cohort comparison, AUC values were numerically lower under charcoal-block than under non-block conditions, whereas C_max_ and T_max_ were generally similar. For the test formulation, mean AUC_0-t_ decreased from 2,135.20 ± 598.52 to 1927.92 ± 543.73 h·pg/mL, and mean AUC_0-inf_ decreased from 2,224.24 ± 613.06 to 2000.58 ± 550.29 h·pg/mL; however, the corresponding nominal exploratory P values were 0.129 and 0.108. Similar patterns were observed for the reference formulation. A sensitivity analysis based on mean log-transformed subject-level summaries yielded the same overall conclusion. These findings are consistent with, but do not establish, a predominant pulmonary contribution to systemic exposure after nebulization. The observed numerical pattern is biologically plausible given the substantial first-pass metabolism of budesonide [6, 15–18], but the separate-cohort design and absence of statistically significant AUC differences require cautious interpretation.

At the same time, this comparison should not be overinterpreted. The non-block and charcoal-block conditions were studied in two separate cohorts rather than within the same participants. Accordingly, the between-study comparison cannot be interpreted as a randomized estimate of the effect of charcoal blockade and should instead be viewed as exploratory supportive evidence. All exploratory P values were nominal because no multiplicity adjustment was applied. We have therefore used associative rather than causal language when discussing the charcoal-related exposure differences.

The concordance between the two BE trials remains important from both pharmacological and regulatory perspectives. The non-block study supports equivalence for total systemic exposure, whereas the charcoal-block study provides complementary, lung-focused pharmacokinetic evidence that systemic exposure was comparable between formulations under conditions intended to reduce gastrointestinal absorption. Together, these findings align with regulatory expectations for the development of locally acting orally inhaled products when gastrointestinal absorption may complicate interpretation [10, 11].

Some statistically significant period and formulation terms were observed in the analysis of variance for the charcoal-block study. These findings should be interpreted within the conventional framework of BE evaluation rather than in isolation. Regulatory BE conclusions are based primarily on the 90% CIs for the GMRs, and all three primary endpoints remained within the prespecified acceptance range.

This study has several methodological strengths, including the replicated four-period crossover design, standardized nebulization training, intensive early PK sampling, and the inclusion of a dedicated charcoal-block trial. These design features improve the sensitivity of the within-study BE assessment and help inform a cautious interpretation of pulmonary versus gastrointestinal contributions to systemic exposure.

Several limitations should also be recognized. First, the charcoal-block and non-block trials were conducted in separate cohorts rather than within the same participants, so the cross-study comparison should be regarded as exploratory. Second, the trials were performed in healthy volunteers rather than in patients with asthma or COPD, and inhalation patterns or airway geometry may differ in clinical populations. Third, only single-dose PK parameters were evaluated; therefore, the present results do not address repeated-dose exposure, accumulation, steady-state pharmacokinetics, or long-term use in patients with chronic airway disease. Fourth, the efficiency of charcoal blockade depended on compliance with gargling and administration instructions. Although these procedures were standardized, they could not directly verify complete suppression of gastrointestinal absorption. Finally, lung deposition may vary with nebulizer system performance and inhalation technique, which may limit extrapolation to other devices.

Future work could extend these findings by integrating PK data with *in vitro* performance testing, deposition modeling, or patient-based studies to better characterize how device, formulation, and inhalation pattern influence lung delivery. Even so, the present trials provide useful clinical pharmacology evidence for the evaluation of generic nebulized budesonide products.

## Conclusion

The test and reference BIS formulations were bioequivalent in healthy Chinese subjects under both non-block and charcoal-block conditions. In the exploratory between-cohort subject-level comparison, activated charcoal was associated with numerically lower AUC values, while C_max_ and T_max_ were similar between conditions; nominal exploratory P values for C_max_, AUC_0-t_, and AUC_0-inf_ did not indicate statistically significant between-cohort differences, and a log-scale sensitivity analysis yielded the same overall conclusion. These findings are consistent with, but do not establish, a predominant pulmonary contribution to systemic budesonide exposure after nebulization. Because the conditions were evaluated in separate cohorts and multiplicity was not adjusted, the charcoal-related findings should be interpreted cautiously. Both formulations were generally well tolerated, and no new safety signal was identified. Together, the two trials provide supportive PK evidence for comparable systemic exposure under both standard and gastrointestinal absorption-reduced conditions.

## Data Availability

The raw data supporting the conclusions of this article will be made available by the authors, without undue reservation.
