# Supplementary material for: Bioequivalence of budesonide inhalation suspension with and without activated charcoal in healthy Chinese subjects: two randomized four-period crossover trials
Source: J Pharm Pharm Sci. 2026 Oct 20;29:17048. doi: 10.3389/jpps.2026.17048 (PMC13727514; doi:10.3389/jpps.2026.17048)
Supplement: Supplementary file 1 [file Supplementaryfile1.docx]

**Supplementary S1**

**Bioanalytical Method Validation Summary for Plasma Budesonide**

**Scope note.** The supplied validation report states that the assay was validated under contemporaneous applicable requirements, including FDA, EMA, Chinese Pharmacopoeia, and GCP references. Because final ICH M10 was adopted after this study, this summary is organized by the validation elements expected in current FDA/ICH M10 reporting; it does not retrospectively claim that the study was conducted under ICH M10.

**S1.1 Assay overview**

Budesonide concentrations in human K2-EDTA plasma were determined using a validated solid-phase extraction LC-MS/MS method. Budesonide-d8 was used as the internal standard. Chromatographic separation was performed on a Waters Acquity UPLC BEH C18 column (2.1 x 50 mm, 1.7 um) with a C18 guard column, followed by negative-electrospray tandem mass-spectrometric detection. The monitored transitions were m/z 489.3→357.1 for budesonide and m/z 497.3→357.0 for budesonide-d8.

# S1.2 Validation summary

| **Validation element** | **Design / condition** | **Verified result** |
| --- | --- | --- |
| Matrix and calibration | Human K2-EDTA plasma. Seven non-zero calibrators: 10.00, 20.00, 60.00, 200, 1000, 1600, and 2000 pg/mL; 1/concentration squared weighted regression. | Calibration range: 10.00-2000 pg/mL; mean r2 across reported validation/activation runs: 0.9983 (range 0.9968-0.9994). |
| LLOQ | 10.00 pg/mL. LLOQ QC (QC1) was evaluated in replicate validation runs. | Within-run CV: 7.14%; bias: -2.47% to 13.60%. Between-run CV: 8.89%; bias: 4.20%. |
| Accuracy and precision | QC2 30.00 pg/mL, QC3 800.0 pg/mL, and QC4 1500 pg/mL. | Within-run CV≤ 4.91%, bias -2.50% to 4.73%. Between-run CV ≤ 4.38%, bias -0.87% to 1.23%. |
| Selectivity | Six individual normal K2-EDTA plasma lots, plus pooled normal, hemolyzed, and lipemic plasma matrices were evaluated. | Endogenous interference: 2.41-13.41% of LLOQ response at the budesonide retention time and 0.19-2.62% at the IS retention time; within the prespecified <20% and <5% criteria, respectively. |
| Analyte/IS cross-interference | Budesonide and budesonide-d8 mutual interference was assessed. | Budesonide effect on IS: 1.05%; IS effect on LLOQ budesonide response: 7.05%; both met the prespecified acceptance criteria. |
| Matrix effect | Eighteen plasma matrices: six normal, six high-fat, three hemolyzed, and three self-prepared lipemic matrices. IS-normalized matrix factors were assessed at QC2 and QC4. | Mean IS-normalized MF: 1.077 (CV 2.53%) at 30.00 pg/mL and 1.033 (CV 1.91%) at 1500 pg/mL. |
| Extraction recovery | Recovery after solid-phase extraction was evaluated at QC2, QC3, and QC4. | Budesonide recovery: 82.54% (30.00 pg/mL), 92.04% (800.0 pg/mL), and 89.69% (1500 pg/mL); overall 88.09% (CV 7.21%). IS recovery: 89.57% (CV 6.66%). |
| Dilution integrity | QC.DIL 6000 pg/mL was diluted sixfold with blank human plasma (n=6). | Mean measured concentration: 5888 pg/mL; CV 1.59%; bias -1.87%. |
| Carryover | Blank samples following upper-limit calibrators were examined during validation. | Analyte carryover 4.66-17.87% of LLOQ response; IS carryover 0-1.32% of IS response; both met the study prespecified criteria. |
| Stability | Plasma stability was assessed after four freeze-thaw cycles; 8 h on ice before processing; 72 h in the autosampler after processing; 78 h reinjection; and long-term storage. Whole blood stability on ice was also assessed. | All tested conditions met acceptance criteria. Long-term plasma stability: 23 d at -30 ℃ to -10℃ -50 ℃ to -30 ℃, and -80 ℃ to -60 ℃; and 74 d at -50 ℃ to -30 ℃ and -80 ℃ to -60 ℃. Whole blood was stable on ice for 2 h. |
| Robustness | Column and analyst crossover assessments were performed. | For QC2-QC4, CV ≤ 5.70% and bias -5.43% to 8.80%; LLOQ QC CV ≤ 11.48% and bias -0.38% to -0.14%. |

# S1.3 Formal-study sample-analysis check

The supplied formal-study sample-analysis report documented incurred-sample reanalysis (ISR) for 288 samples. All 288 ISR results met the study acceptance criteria (100%). This result pertains to the supplied formal-study report and is presented as a supporting sample-analysis quality check, rather than as a replacement for the primary validation data above.

# S1.4 Source and reporting note

All numerical values in this supplement were transcribed from the supplied method-validation report (project 19-229) and the supplied formal-study sample-analysis report. The analytical method was originally documented with references to contemporaneous FDA and EMA bioanalytical-validation guidance and other applicable requirements. The table layout above maps the reported evidence to the validation domains emphasized by current FDA guidance and ICH M10.
